# Decision Trees for Binary Subword-Closed Languages

**DOI:** 10.3390/e25020349

**Published:** 2023-02-14

**Authors:** Mikhail Moshkov

**Affiliations:** Computer, Electrical and Mathematical Sciences & Engineering Division and Computational Bioscience Research Center, King Abdullah University of Science and Technology (KAUST), Thuwal 23955-6900, Saudi Arabia; mikhail.moshkov@kaust.edu.sa

**Keywords:** subword-closed language, recognition problem, membership problem, deterministic decision tree, nondeterministic decision tree

## Abstract

In this paper, we study arbitrary subword-closed languages over the alphabet {0,1} (binary subword-closed languages). For the set of words L(n) of the length *n* belonging to a binary subword-closed language *L*, we investigate the depth of the decision trees solving the recognition and the membership problems deterministically and nondeterministically. In the case of the recognition problem, for a given word from L(n), we should recognize it using queries, each of which, for some i∈{1,…,n}, returns the *i*th letter of the word. In the case of the membership problem, for a given word over the alphabet {0,1} of the length *n*, we should recognize if it belongs to the set L(n) using the same queries. With the growth of *n*, the minimum depth of the decision trees solving the problem of recognition deterministically is either bounded from above by a constant or grows as a logarithm, or linearly. For other types of trees and problems (decision trees solving the problem of recognition nondeterministically and decision trees solving the membership problem deterministically and nondeterministically), with the growth of *n*, the minimum depth of the decision trees is either bounded from above by a constant or grows linearly. We study the joint behavior of the minimum depths of the considered four types of decision trees and describe five complexity classes of binary subword-closed languages.

## 1. Introduction

In this paper, we study arbitrary binary languages (languages over the alphabet E={0,1}) that are subword closed: if a word $w_{1}u_{1}w_{2}\cdots w_{m}u_{m}w_{m+1}$ belongs to a language, then the word $u_{1}\cdots u_{m}$ belongs to this language. Subword-closed languages have attracted the attention of researchers in the field of formal languages for many years [1,2,3,4,5].

For the set of words L(n) of the length *n* belonging to a binary subword-closed language *L*, we investigate the depth of the decision trees solving the recognition and the membership problems deterministically and nondeterministically. In the case of the recognition problem, for a given word from L(n), we should recognize it using queries, each of which, for some i∈{1,…,n}, returns the *i*th letter of the word. In the case of the membership problem, for a given word over the alphabet *E* of the length *n*, we should recognize if it belongs to L(n) using the same queries.

For an arbitrary binary subword-closed language, with the growth of *n*, the minimum depth of the decision trees solving the problem of recognition deterministically is either bounded from above by a constant or grows as a logarithm, or linearly. For other types of trees and problems (decision trees solving the problem of recognition nondeterministically and decision trees solving the membership problem deterministically and nondeterministically), with the growth of *n*, the minimum depth of decision trees is either bounded from above by a constant or grows linearly. We study the joint behavior of the minimum depths of the considered four types of decision trees and describe five complexity classes of binary subword-closed languages.

In [6], the following results were announced without proof. For an arbitrary regular language, with the growth of *n*, (i) the minimum depth of the decision trees solving the problem of recognition deterministically is either bounded from above by a constant or grows as a logarithm, or linearly, and (ii) the minimum depth of the decision trees solving the problem of recognition nondeterministically is either bounded from above by a constant or grows linearly. Proofs for the case of decision trees solving the problem of recognition deterministically can be found in [7,8]. To apply the considered results to a given regular language, it is necessary to know a deterministic finite automaton (DFA) accepting this language.

Each subword-closed language over a finite alphabet is a regular language [3]. In this paper, we do not assume that binary subword-closed languages are given by DFAs. So, we cannot use the results from [6,7,8]. Instead of this, for binary subword-closed languages, we describe simple criteria for the behavior of the minimum depths of decision trees solving the problems of recognition and membership deterministically and nondeterministically.

This paper is a theoretical work related to the field of formal languages. It has no direct applications. In the theory of formal languages, various parameters of languages are studied, in particular the growth of the number of words of the language with the growth of the length of words and, for regular languages, the minimum number of states of the automaton accepting the language. For many years, the author has been introducing new parameters of languages into scientific use: the minimum depth of deterministic and nondeterministic decision trees for the recognition and membership problems related to the language [6,7,8,9]. The present paper continues this line of research.

There is now an extensive collection of methods for constructing decision trees. It includes (i) a variety of greedy heuristics based on measures of uncertainty, such as entropy and the Gini index [10,11,12], (ii) exact optimization algorithms based on dynamic programming, branch-and-bound search, SAT-based methods, etc., [13,14,15,16], and (iii) approximate optimization algorithms with bounds of accuracy that are applicable to obtain theoretical results about the complexity of decision trees [8,17].

In this paper, we found simple combinatorial parameters of binary subword-closed languages, which made it possible to obtain bounds on the depth of the decision trees without using the effective but rather complicated methods developed in the monographs [8,17].

The rest of this paper is organized as follows. In Section 2, we consider the main notions; in Section 3, the main results; in Section 4, the proofs; and in Section 5, short conclusions.

## 2. Main Notions

Let ω={0,1,2,…} be the set of nonnegative integers and E={0,1}. By $E^{*}$, we denote the set of all finite words over the alphabet *E*, including the empty word λ. Any subset *L* of the set $E^{*}$ is called a binary language. This language is called subword closed if, for any word $w_{1}u_{1}w_{2}\cdots w_{m}u_{m}w_{m+1}$ belonging to *L*, the word $u_{1}\cdots u_{m}$ belongs to *L*, where $w_{i}$, $u_{j}$$\in E^{*}$, i=1,…,m+1, j=1,…,m. For any natural *n*, we denote by L(n) the set of words from *L*, for which length is equal to *n*. We consider two problems related to the set L(n). The problem of recognition: for a given word from L(n), we should recognize it using attributes (queries) $l_{1}^{n},\ldots ,l_{n}^{n}$, where $l_{i}^{n}$, i∈{1,…,n}, is a function from $E^{*}\left(n\right)$ to *E* such that $l_{i}^{n}\left(a_{1}\cdots a_{n}\right)=a_{i}$ for any word $a_{1}\cdots a_{n}\in E^{*}\left(n\right)$. The problem of membership: for a given word from $E^{*}\left(n\right)$, we should recognize if this word belongs to the set L(n) using the same attributes. To solve these problems, we use decision trees over L(n).

A decision tree over L(n) is a marked finite directed tree with the root, which has the following properties:

- The root and the edges leaving the root are not labeled.
- Each node, which is not the root or terminal node, is labeled with an attribute from the set $\left\{l_{1}^{n},\ldots ,l_{n}^{n}\right\}$.
- Each edge leaving a node, which is not a root, is labeled with a number from *E*.

A decision tree over L(n) is called deterministic if it satisfies the following conditions:

- Exactly one edge leaves the root.
- For any node, which is not the root nor terminal node, the edges leaving this node are labeled with pairwise different numbers.

Let Γ be a decision tree over L(n). *A* complete path in Γ is any sequence $\xi =v_{0},e_{0},\ldots ,v_{m},e_{m},v_{m+1}$ of nodes and edges of Γ such that $v_{0}$ is the root, $v_{m+1}$ is a terminal node, $v_{i}$ is the initial, and $v_{i+1}$ is the terminal node of the edge $e_{i}$ for i=0,…,m. We define a subset E(n,ξ) of the set $E^{*}\left(n\right)$ in the following way: if m=0, then $E\left(n,\xi \right)=E^{*}\left(n\right)$. Let m>0, the attribute $l_{i_{j}}^{n}$ be assigned to the node $v_{j}$ and $b_{j}$ be the number assigned to the edge $e_{j}$, j=1,…,m. Then,

$$E\left(n,\xi \right)=\{a_{1}\cdots a_{n}\in E^{*}\left(n\right):a_{i_{1}}=b_{1},\ldots ,a_{i_{m}}=b_{m}\}.$$

Let L(n)≠∅. We say that a decision tree Γ over L(n) solves the problem of recognition for L(n) nondeterministically if Γ satisfies the following conditions:

- Each terminal node of Γ is labeled with a word from L(n).
- For any word w∈L(n), there exists a complete path ξ in the tree Γ such that w∈E(n,ξ).
- For any word w∈L(n) and for any complete path ξ in the tree Γ such that w∈E(n,ξ), the terminal node of the path ξ is labeled with the word *w*.

We say that a decision tree Γ over L(n) solves the problem of recognition for L(n) deterministically if Γ is a deterministic decision tree, which solves the problem of recognition for L(n) nondeterministically.

We say that a decision tree Γ over L(n) solves the problem of membership for L(n) nondeterministically if Γ satisfies the following conditions:

- Each terminal node of Γ is labeled with a number from *E*.
- For any word $w\in E^{*}\left(n\right)$, there exists a complete path ξ in the tree Γ such that w∈E(n,ξ).
- For any word $w\in E^{*}\left(n\right)$ and for any complete path ξ in the tree Γ such that w∈E(n,ξ), the terminal node of the path ξ is labeled with the number 1 if w∈L(n) and with the number 0, otherwise.

We say that a decision tree Γ over L(n) solves the problem of membership for L(n) deterministically if Γ is a deterministic decision tree which solves the problem of membership for L(n) nondeterministically.

Let Γ be a decision tree over L(n). We denote by h(Γ) the maximum number of nodes in a complete path in Γ that are not the root nor terminal node. The value h(Γ) is called the depth of the decision tree Γ.

We denote by $h_{L}^{ra}\left(n\right)$ ($h_{L}^{rd}\left(n\right)$) the minimum depth of a decision tree, which solves the problem of recognition for L(n) nondeterministically (deterministically). If L(n)=∅, then $h_{L}^{ra}\left(n\right)=h_{L}^{rd}\left(n\right)=0$.

We denote by $h_{L}^{ma}\left(n\right)$ ($h_{L}^{md}\left(n\right)$) the minimum depth of a decision tree, which solves the problem of membership for L(n) nondeterministically (deterministically). If L(n)=∅, then $h_{L}^{ma}\left(n\right)=h_{L}^{md}\left(n\right)=0$.

## 3. Main Results

Let *L* be a binary subword-closed language. For any a∈E and i∈ω, we denote by $a^{i}$ the word a⋯a of the length *i* (if i=0, then $a^{i}=\lambda$). For any a∈E, let $\bar{a}=1$ if a=0 and $\bar{a}=0$ if a=1.

We define the parameter Hom(L) of the language *L*, which is called the homogeneity dimension of the language *L*. If for each natural number *m*, there exists a∈E such that the word $a^{m}\bar{a}a^{m}$ belongs to *L*, then Hom(L)=∞. Otherwise, Hom(L) is the maximum number m∈ω such that there exists a∈E for which the word $a^{m}\bar{a}a^{m}$ belongs to *L*. If L=∅, then Hom(L)=0.

We now define the parameter Het(L) of the language *L*, which is called the heterogeneity dimension of the language *L*. If for each natural number *m*, there exists a∈E such that the word $a^{m}{\bar{a}}^{m}$ belongs to *L*, then Het(L)=∞. Otherwise, Het(L) is the maximum number m∈ω such that there exists a∈E for which the word $a^{m}{\bar{a}}^{m}$ belongs to *L*. If L=∅, then Het(L)=0.

**Theorem 1.**  *Let L be a binary subword-closed language.*

*(a)*  * If Hom(L)=∞, then $h_{L}^{rd}\left(n\right)=\Theta \left(n\right)$ and $h_{L}^{ra}\left(n\right)=\Theta \left(n\right)$.*
*(b)*  *If Hom(L)<∞ and Het(L)=∞, then $h_{L}^{rd}\left(n\right)=\Theta \left(\log n\right)$ and $h_{L}^{ra}\left(n\right)=O\left(1\right)$.*
*(c)*  *If Hom(L)<∞ and Het(L)<∞, then $h_{L}^{rd}\left(n\right)=O\left(1\right)$ and $h_{L}^{ra}\left(n\right)=O\left(1\right)$.*

**Example 1.**  *Let us consider the binary subword-closed language $L_{0}=\left\{1^{i}0^{j}:i,j\in \omega \right\}$. One can show that $Hom(L_{0})=0$ and $Het(L_{0})=\infty$. By Theorem 1, $h_{L_{0}}^{rd}\left(n\right)=\Theta \left(\log n\right)$ and $h_{L_{0}}^{ra}\left(n\right)=O\left(1\right)$.*

For a binary subword-closed language *L*, we denote by $L^{C}$ its complementary language $E^{*}\setminus L$. The notation |L|=∞ means that *L* is an infinite language, and the notation |L|<∞ means that *L* is a finite language.

**Theorem 2.**  *Let L be a binary subword-closed language.*

*(a)*  * If |L|=∞ and $L^{C}\neq \emptyset$, then $h_{L}^{md}\left(n\right)=\Theta \left(n\right)$ and $h_{L}^{ma}\left(n\right)=\Theta \left(n\right)$.*
*(b)*  * If |L|<∞ or $L^{C}=\emptyset$, then $h_{L}^{md}\left(n\right)=O\left(1\right)$ and $h_{L}^{ma}\left(n\right)=O\left(1\right)$.*

**Example 2.**  *One can show that, for the binary subword-closed language $L_{0}=\left\{1^{i}0^{j}:i,j\in \omega \right\}$, considered in Example 1, $|L_{0}|=\infty$ and $L_{0}^{C}\neq \emptyset$. By Theorem 2, $h_{L_{0}}^{md}\left(n\right)=\Theta \left(n\right)$ and $h_{L_{0}}^{ma}\left(n\right)=\Theta \left(n\right)$.*

To study all possible types of joint behavior of functions $h_{L}^{rd}\left(n\right)$, $h_{L}^{ra}\left(n\right)$, $h_{L}^{md}\left(n\right)$, and $h_{L}^{ma}\left(n\right)$ for binary subword-closed languages *L*, we consider five classes of languages $L_{1},\ldots ,L_{5}$ described in the columns 2–5 of Table 1. In particular, $L_{1}$ consists of all binary subword-closed languages *L* with Hom(L)=∞and $L^{C}\neq \emptyset$. It is easy to show that the complexity classes $L_{1},\ldots ,L_{5}$ are pairwise disjointed, and each binary subword-closed language belongs to one of these classes. The behavior of functions $h_{L}^{rd}\left(n\right)$, $h_{L}^{ra}\left(n\right)$, $h_{L}^{md}\left(n\right)$, and $h_{L}^{ma}\left(n\right)$ for languages from these classes is described in the last four columns of Table 1. For each class, the results considered in Table 1 follow from Theorems 1 and 2 and the following three remarks: (i) from the condition Hom(L)=∞, it follows |L|=∞, (ii) from the condition Het(L)=∞, it follows |L|=∞, and (iii) from the condition Hom(L)<∞, it follows $L^{C}\neq \emptyset$.

We now show that the classes $L_{1},\ldots ,L_{5}$ are nonempty. To this end, we consider the following five binary subword-closed languages:

$$\begin{array}{ccc} L_{1} & = & \{0^{i}10^{j},0^{i}:i,j\in \omega \},\\ L_{2} & = & E^{*},\\ L_{3} & = & \{0^{i}1^{j}:i,j\in \omega \},\\ L_{4} & = & \{0^{i}:i\in \omega \},\\ L_{5} & = & \{0\}. \end{array}$$

It is easy to see that $L_{i}\in L_{i}$ for i=1,…,5.

## 4. Proofs of Theorems 1 and 2

In this section, we prove Theorems 1 and 2. First, we consider two auxiliary statements. For a word w, we denote by |w| its length.

**Lemma 1.**  *Let L be a binary subword-closed language for which Hom(L)<∞. Then, any word w from L can be represented in the form*

(1) $$w_{1}a^{i}w_{2}{\bar{a}}^{j}w_{3},$$

where a∈E, i,j∈ω, and $w_{1}$, $w_{2}$, $w_{3}$ are words from $E^{*}$ with length at most 2Hom(L) each.

**Proof.**  Denote m=Hom(L). Then, the words $0^{m+1}10^{m+1}$ and $1^{m+1}01^{m+1}$ do not belong to *L*. Let *w* be a word from *L*. Then, for any a∈E, any entry of the letter *a* in *w* has at most *m*$\bar{a}$s to the left of this entry (we call it *l*-entry of *a*) or at most *m*$\bar{a}$s to the right of this entry (we call it *r*-entry of *a*). Let a∈E. We say that *w* is (i) *a*-*l*-word if any entry of *a* in *w* is *l*-entry; (ii) *a*-*r*-word if any entry of *a* in *w* is *r*-entry; and (iii) *a*-*b*-word if *w* is not *a*-*l*-word and is not *a*-*r*-word. Let c,d∈{l,r,b}. We say that *w* is cd-word if *w* is 0-*c*-word and 1-*d*-word. There are nine possible pairs cd. We divide them into four groups: (a) ll and rr, (b) lr and rl, (c) lb, rb, bl, and br, and (d) bb, and consider them separately. Let

$$w=a_{1}\cdots a_{n}.$$

We assume that *w* contains both 0s and 1s. Otherwise, *w* can be represented in the form (1).

(a) Let *w* be ll-word. Let $a_{n}=0$ and $a_{i}$ be the rightmost entry of 1 in *w*. Because *w* is ll-word, there are at most *m* 1s to the left of $a_{n}$ and at most *m* 0s to the left of $a_{i}$. Denote $w_{1}=a_{1}\cdots a_{i}$. Then, $w_{1}$ contains at most *m* 0s and at most *m* 1s, i.e., the length of $w_{1}$ is at most 2m. Moreover, to the right of $a_{i}$, there are only 0s. Thus, $w=w_{1}0^{n-i}$, where $|w_{1}|=i\leq 2m$, i.e., *w* can be represented in the form (1).

Let $a_{n}=1$ and $a_{i}$ be the rightmost entry of 0 in *w*. Denote $w_{1}=a_{1}\cdots a_{i}$. Then, $w_{1}$ contains at most *m* 0s and at most *m* 1s, i.e., $|w_{1}|\leq 2m$. Moreover, to the right of $a_{i}$, there are only 1s. Thus, $w=w_{1}1^{n-i}$, i.e., *w* can be represented in the form (1).

One can prove in a similar way that any rr-word can be represented in the form (1).

(b) Let *w* be lr-word, $a_{i}$ be the rightmost entry of 0, and $a_{j}$ be the leftmost entry of 1. Then, either j=i+1 or j<i. Let j=i+1. Then, $w=0^{i}1^{n-i}$, i.e., *w* can be represented in the form (1). Let now j<i. Denote $w_{2}=a_{j}\cdots a_{i}$. The word *w* has at most *m* 0s to the right of $a_{j}$ and at most *m* 1s to the left of $a_{i}$. Therefore, $|w_{2}|\leq 2m$ and $w=0^{j-1}w_{2}1^{n-i}$, i.e., *w* can be represented in the form (1).

One can prove in a similar way that any rl-word can be represented in the form (1).

(c) Let *w* be lb-word; $a_{i}$ be the rightmost entry of 1 such that to the left of this entry, we have at most *m* 0s; and $a_{j}$ be the next after $a_{i}$ entry of 1. It is clear that to the right of $a_{j}$, there are at most *m* 0s, j≥i+2, and all letters $a_{i+1},\ldots ,a_{j-1}$ are equal to 0. Let $a_{k}$ be the rightmost entry of 0. Then, to the left of $a_{k}$, there are at most *m* 1s. It is clear that either k=j−1 or k>j. Denote $w_{1}=a_{1}\cdots a_{i}$. Then, $|w_{1}|\leq 2m$. Let k=j−1. In this case, $w=w_{1}0^{j-i-1}1^{n-j+1}$, i.e., *w* can be represented in the form (1). Let k>j. Denote $w_{2}=a_{j}\cdots a_{k}$. Then, $|w_{2}|\leq 2m$. We have $w=w_{1}0^{j-i-1}w_{2}1^{n-k}$, i.e., *w* can be represented in the form (1).

One can prove in a similar way that any rb- or bl- or br-word can be represented in the form (1).

(d) Let *w* be bb-word, $a_{i}$ be the rightmost entry of 0 such that there are at most *m* 1s to the left of this entry, and $a_{j}$ be the next after $a_{i}$ entry of 0. Then, there are at most *m* 1s to the right of $a_{j}$, j≥i+2, and $w=a_{1}\cdots a_{i}1\cdots 1a_{j}\cdots a_{n}$. Denote A={1,…,i}, B={i+1,…,j−1}, and C={j,…,n}. Let $a_{k}$ be the rightmost entry of 1 such that there are at most *m* 0s to the left of this entry and $a_{l}$ be the next after $a_{k}$ entry of 1. Then, there are at most *m* 0s to the right of $a_{l}$, l≥k+2, and $w=a_{1}\cdots a_{k}0\cdots 0a_{l}\cdots a_{n}$.

There are four possible types of location of $a_{k}$ and $a_{l}$: (i) k∈A and l∈A, (ii) k∈A and l∈B (the combination k∈A and l∈C is impossible because all letters with indices from *B* are 1s, but all letters between $a_{k}$ and $a_{l}$ are 0s), (iii) k∈B and l∈C (the combination k∈B and l∈B is impossible because all letters with indices from *B* are 1s, but all letters between $a_{k}$ and $a_{l}$ are 0s), and (iv) k∈C and l∈C. We now consider cases (i)–(iv) in detail.

(i) Let k∈A and l∈A. Then, $w=a_{1}\cdots a_{k}0\cdots 0a_{l}\cdots a_{i}1\cdots 1a_{j}\cdots a_{n}$. Denote $w_{1}=a_{1}\cdots a_{k}$, $w_{2}=a_{l}\cdots a_{i}$, and $w_{3}=a_{j}\cdots a_{n}$. The length of $w_{1}$ is at most 2m because from the left of $a_{k}$, there are at most *m* 0s, and from the left of $a_{i}$, there are at most *m* 1s. We can prove in a similar way that $|w_{2}|\leq 2m$ and $|w_{3}|\leq 2m$. Therefore, *w* can be represented in the form (1).

(ii) Let k∈A and l∈B. Then, l=i+1 and

$$w=a_{1}\cdots a_{k}0\cdots 0a_{i}a_{i+1}1\cdots 1a_{j}\cdots a_{n},$$

where $a_{i}=0$ and $a_{i+1}=1$. Denote $w_{1}=a_{1}\cdots a_{k}$ and $w_{3}=a_{j}\cdots a_{n}$. It is easy to show that $|w_{1}|\leq 2m$ and $|w_{3}|\leq 2m$. Therefore, *w* can be represented in the form (1).

(iii) Let k∈B and l∈C. Then, k=j−1 and

$$w=a_{1}\cdots a_{i}1\cdots 1a_{j-1}a_{j}0\cdots 0a_{l}\cdots a_{n},$$

where $a_{j-1}=1$ and $a_{j}=0$. Denote $w_{1}=a_{1}\cdots a_{i}$ and $w_{3}=a_{l}\cdots a_{n}$. It is easy to show that $|w_{1}|\leq 2m$ and $|w_{3}|\leq 2m$. Therefore, *w* can be represented in the form (1).

(iv) Let k∈C and l∈C. Then, $w=a_{1}\cdots a_{i}1\cdots 1a_{j}\cdots a_{k}0\cdots 0a_{l}\cdots a_{n}$. Denote $w_{1}=a_{1}\cdots a_{i}$, $w_{2}=a_{j}\cdots a_{k}$, and $w_{3}=a_{l}\cdots a_{n}$. It is easy to show that $|w_{1}|\leq 2m$, $|w_{2}|\leq 2m$, and $|w_{3}|\leq 2m$. Therefore, *w* can be represented in the form (1). □

**Lemma 2.**  *Let L be a binary subword-closed language for which Hom(L)<∞ and Het(L)<∞. Then, there exists natural p such that |L(n)|≤p for any natural n.*

**Proof.**  Denote m=max(Hom(L),Het(L)). Then, the words $0^{m+1}1^{m+1}$ and $1^{m+1}0^{m+1}$ do not belong to *L*. Using Lemma 1, we obtain that each word *w* from *L* can be represented in the form $w_{1}a^{i}w_{2}{\bar{a}}^{j}w_{3}$, where a∈E, the length of $w_{k}$ is at most t=2m for k=1,2,3, i,j∈ω, and i≤m or j≤m. We now evaluate the number of such words, for which length is equal to *n*. Let k∈{1,2,3}. Then, the number of different words $w_{k}$ is at most $2^{0}+2^{1}+\cdots +2^{t}<2^{t+1}$. Let us assume that the words $w_{1}$, $w_{2}$, and $w_{3}$ are fixed and $|w_{1}\left|+\right|w_{2}\left|+\right|w_{3}|\leq n$. Then, the number of different words $a^{i}{\bar{a}}^{j}$ of the length $n-|w_{1}\left|-\right|w_{2}\left|-\right|w_{3}|$ is at most 4(m+1) because i≤m or j≤m. Thus, the number of words in L(n) is at most $p=2^{3t+3}\left(2t+4\right)$. □

**Proof of Theorem 1.**  It is clear that $h_{L}^{ra}\left(n\right)\leq h_{L}^{rd}\left(n\right)$ for any natural *n*.

(a) Let Hom(L)=∞ and *n* be a natural number. Then, there exists a∈E such that $a^{n}\bar{a}a^{n}\in L$. Therefore, $a^{n},a^{i}\bar{a}a^{n-i-1}\in L\left(n\right)$ for i=0,…,n−1. Let Γ be a decision tree over L(n), which solves the problem of recognition for L(n) nondeterministically and has the minimum depth $h_{L}^{ra}\left(n\right)$, and ξ be a complete path in Γ such that $a^{n}\in E\left(n,\xi \right)$. Let us assume that there is i∈{0,…,n−1} such that the attribute $l_{i+1}^{n}$ is not attached to any node of ξ, which is not the root nor the terminal node. Then, $a^{i}\bar{a}a^{n-i-1}\in E\left(n,\xi \right)$, which is impossible. Therefore, h(Γ)≥n and $h_{L}^{ra}\left(n\right)\geq n$. It is easy to show that $h_{L}^{rd}\left(n\right)\leq n$. Thus, $h_{L}^{ra}\left(n\right)=h_{L}^{rd}\left(n\right)=n$ for any natural *n*.

(b) Let Hom(L)<∞ and Het(L)=∞. By Lemma 1, each word from *L* can be represented in the form $w_{1}a^{i}w_{2}{\bar{a}}^{j}w_{3}$, where a∈E, the length of $w_{k}$ is at most t=2Hom(L) for k=1,2,3, and i,j∈ω. Note that either $w_{2}=\lambda$ or $w_{2}$ is a word of the kind $\bar{a}\cdots a$.

Let *n* be a natural number such that n≥10t. We now describe the work of a decision tree over L(n), which solves the problem of recognition for L(n) deterministically. Let w∈L(n). We represent this word as follows: $w=L_{1}L_{2}L_{3}AR_{3}R_{2}R_{1}$, where the length of each word $L_{1},L_{2},L_{3},R_{3},R_{2},R_{1}$ is equal to *t*. First, we recognize all letters in the words $L_{1},L_{2},R_{2},R_{1}$ using 4t queries (attributes). We now consider four cases.

(i) Let $L_{2}=R_{2}=a^{t}$ for some a∈E. Then, $L_{3}AR_{3}=a^{n-4t}$, and the word *w* is recognized.

(ii) Let $L_{2}=a^{t}$ for some a∈E, and $R_{2}$ contains both 0 and 1. Then, $R_{2}$ has an intersection with the word $w_{2}$. It is clear that $w_{2}$ has no intersection with the word *A* and $L_{3}A=a^{n-5t}$. We recognize all letters of the word $R_{3}$. As a result, the word *w* will be recognized.

(iii) Let $R_{2}=a^{t}$ for some a∈E, and $L_{2}$ contains both 0 and 1. Then, $L_{2}$ has an intersection with the word $w_{2}$. It is clear that $w_{2}$ has no intersection with the word *A* and $AR_{3}=a^{n-5t}$. We recognize all letters of the word $L_{3}$. As a result, the word *w* will be recognized.

(iv) Let $L_{2}=a^{t}$ and $R_{2}={\bar{a}}^{t}$ for some a∈E. Then, we need to recognize the position of the word $w_{2}$ and the word $w_{2}$ itself. Beginning with the left, we divide $L_{3}AR_{3}$ and, probably, a prefix of $R_{2}$ into blocks of the length *t*. As a result, we have k≤n/t blocks. We recognize all letters in the block with the number $r=\left\lceil k/2\right\rceil$. If all letters in this block are equal to $\bar{a}$, then we apply the same procedure to the blocks with numbers 1,…,r−1. If all letters in this block are equal to *a*, then we apply the same procedure to the blocks with numbers r+1,…,k. If the considered block contains both 0 and 1, then we recognize *t* letters before this block and *t* letters after this block and, as a result, recognize both the word $w_{2}$ and its position. After each iteration, the number of blocks is at most one-half of the previous number of blocks. Let *q* be the whole number of iterations. Then, after the iteration q−1, we have at least one unchecked block. Therefore, $k/2^{q-1}\geq 1$ and $q\leq \log_{2}k+1$.

In case (i), to recognize the word *w*, we make 4t queries. In cases (ii) and (iii), we make 5t queries. In case (iv), we make at most $t\log_{2}\left(n/t\right)+7t$ queries. As a result, we have $h_{L}^{rd}\left(n\right)=O\left(\log n\right)$.

Because Het(L)=∞, for any natural *n*, the set L(n) contains for some a∈E words $a^{i}{\bar{a}}^{n-i}$ for i=0,…,n. Then, |L(n)|≥n+1, and each decision tree Γ over L(n) solving the problem of recognition for L(n) deterministically has at least n+1 terminal nodes. One can show that the number of terminal nodes in Γ is at most $2^{h(\Gamma )}$. Therefore, $h\left(\Gamma \right)\geq \log_{2}\left(n+1\right)$. Thus, $h_{L}^{rd}\left(n\right)=\Omega \left(\log n\right)$ and $h_{L}^{rd}\left(n\right)=\Theta \left(\log n\right)$.

We now prove that $h_{L}^{ra}\left(n\right)=O\left(1\right)$. To this end, it is enough to show that there is a natural number *c* such that, for each natural *n* and for each word w∈L(n), there exists a subset $B_{w}$ of the set of attributes $\left\{l_{1}^{n},\ldots ,l_{n}^{n}\right\}$ such that $\left|B_{w}\right|\leq c$ and, for any word u∈L(n) different from *w*, there exists an attribute $l_{i}^{n}\in B_{w}$ for which $l_{i}^{n}\left(w\right)\neq l_{i}^{n}\left(u\right)$. We now show that as *c*, we can use the number 7t. In case (i), in the capacity of the set $B_{w}$, we can choose all attributes corresponding to 4t letters from the subwords $L_{1}$, $L_{2}$, $R_{2}$, and $R_{1}$. In case (ii), we can choose all attributes corresponding to 5t letters from the subwords $L_{1}$, $L_{2}$, $R_{3}$, $R_{2}$, and $R_{1}$. In case (iii), we can choose all attributes corresponding to 5t letters from the subwords $L_{1}$, $L_{2}$, $L_{3}$, $R_{2}$, and $R_{1}$. In case (iv), in the capacity of the set $B_{w}$, we can choose all attributes corresponding to 4t letters from the subwords $L_{1}$, $L_{2}$, $R_{2}$, and $R_{1}$, and 3t letters from the block containing both 0 and 1 and from the blocks that are its left and right neighbors.

(c) Let Hom(L)<∞ and Het(L)<∞. By Lemma 2, there exists natural *p* such that |L(n)|≤p for any natural *n*. Let *n* be a natural number. Then, the set L(n) contains at most *p* words, and there exists a subset *B* of the set of attributes $\left\{l_{1}^{n},\ldots ,l_{n}^{n}\right\}$ such that $\left|B\right|\leq p^{2}$ and, for any two different words u,w∈L(n), there exists an attribute $l_{i}^{n}\in B$ for which $l_{i}^{n}\left(w\right)\neq l_{i}^{n}\left(u\right)$. It is easy to construct a decision tree over L(n) which solves the problem of recognition for L(n) deterministically by sequentially computing attributes from *B*. The depth of this tree is at most $p^{2}$. Therefore, $h_{L}^{rd}\left(n\right)=O\left(1\right)$ and $h_{L}^{ra}\left(n\right)=O\left(1\right)$. □

**Proof of Theorem 2.**  It is clear that $h_{L}^{ma}\left(n\right)\leq h_{L}^{md}\left(n\right)$ for any natural *n*.

(a) Let |L|=∞, $L^{C}\neq \emptyset$, and $w_{0}$ be a word with the minimum length from $L^{C}$. Because |L|=∞, L(n)≠∅ for any natural *n*. Let *n* be a natural number such that $n>|w_{0}|$ and Γ be a decision tree over L(n) that solves the problem of membership for L(n) nondeterministically and has the minimum depth. Let w∈L(n) and ξ be a complete path in Γ such that w∈E(n,ξ). Then, the terminal node of ξ is labeled with the number 1. Let us assume that the number of nodes labeled with attributes in ξ is at most $n-|w_{0}|$. Then, we can change at most $|w_{0}|$ letters in the word *w* such that the obtained word $w^{\prime }$ will satisfy the following conditions: $w_{0}$ is a subword of $w^{\prime }$ and $w^{\prime }\in$E(n,ξ). However, it is impossible because in this case $w^{\prime }\notin L\left(n\right)$ and $w^{\prime }\in$ E(n,ξ), but the terminal node of ξ is labeled with the number 1. Therefore, the depth of Γ is greater than $n-|w_{0}|$. Thus, $h_{L}^{ma}\left(n\right)=\Omega \left(n\right)$. It is easy to construct a decision tree over L(n) that solves the problem of membership for L(n) deterministically and has a depth equal to *n*. Therefore, $h_{L}^{md}\left(n\right)=O\left(n\right)$. Thus, $h_{L}^{md}\left(n\right)=\Theta \left(n\right)$ and $h_{L}^{ma}\left(n\right)=\Theta \left(n\right)$.

(b) Let |L|<∞. Then, there exists natural *m* such that L(n)=∅ for any natural n≥m. Therefore, for each natural n≥m, $h_{L}^{md}\left(n\right)=0$ and $h_{L}^{ma}\left(n\right)=0$.

Let $L^{C}=\emptyset$, *n* be a natural number, and Γ be a decision tree over L(n) which consists of the root, a terminal node labeled with 1, and an edge that leaves the root and enters the terminal node. One can show that Γ solves the problem of membership for L(n) deterministically and has a depth equal to 0. Therefore, $h_{L}^{md}\left(n\right)=0$ and $h_{L}^{ma}\left(n\right)=0$. □

## 5. Conclusions

In this paper, we studied arbitrary binary subword-closed languages. For the set of words L(n) of the length *n* belonging to a binary subword-closed language *L*, we investigated the depth of the decision trees solving the recognition and the membership problems deterministically and nondeterministically. We proved that with the growth of *n*, the minimum depth of the decision trees solving the problem of recognition deterministically is either bounded from above by a constant or grows as a logarithm, or linearly. For other types of trees and problems, with the growth of *n*, the minimum depth of the decision trees is either bounded from above by a constant or grows linearly. We also studied the joint behavior of the minimum depths of the considered four types of decision trees and described five complexity classes of binary subword-closed languages.

In this paper, we did not assume that a binary subword-closed language is given by a deterministic finite automaton accepting this language. So, we could not use the parameters of the automaton for the study of decision tree complexity as it was done in [6,7,8,9]. Instead of this, for binary subword-closed languages, we described simple combinatorial criteria for the behavior of the minimum depths of the decision trees solving the problems of recognition and membership deterministically and nondeterministically.

In the future, we are planning to generalize this approach to some other classes of formal languages.

## Figures and Tables

**Table 1:** Joint behavior of functions $h_{L}^{rd}$, $h_{L}^{ra}$, $h_{L}^{md}$, and $h_{L}^{ma}$ for binary subword-closed languages.

	Hom(L)	Het(L)	|L|	$L^{C}$	$h_{L}^{\mathrm{rd}}$	$h_{L}^{\mathrm{ra}}$	$h_{L}^{\mathrm{md}}$	$h_{L}^{\mathrm{ma}}$
$L_{1}$	=∞			≠∅	Θ(n)	Θ(n)	Θ(n)	Θ(n)
$L_{2}$	=∞			=∅	Θ(n)	Θ(n)	O(1)	O(1)
$L_{3}$	<∞	=∞			Θ(logn)	O(1)	Θ(n)	Θ(n)
$L_{4}$	<∞	<∞	=∞		O(1)	O(1)	Θ(n)	Θ(n)
$L_{5}$	<∞	<∞	<∞		O(1)	O(1)	O(1)	O(1)

## Data Availability

Not applicable.
